# Supplementary material for: The sudden transition to online learning: Teachers’ experiences of teaching during the COVID-19 pandemic
Source: PLoS One. 2023 Nov 16;18(11):e0287520. doi: 10.1371/journal.pone.0287520 (PMC10653415; doi:10.1371/journal.pone.0287520)
Supplement: Attachment — Submitted filename: Response to Reviewers.docx [file pone.0287520.s001.docx]

Additional Editor Comments:

This manuscript is well-written. Improvement in methods section is suggested:

1- Theme network resulting from three-stage coding
2- Table of agreement between two coders or calculation of kappa coefficient
3- The interview questions should come in the tools and methods section


Reviewers' comments:

Reviewer's Responses to Questions

**Comments to the Author**

We thank the reviewer for his/her valuable comments. These comments will improve our manuscript.

1. Is the manuscript technically sound, and do the data support the conclusions?

The manuscript must describe a technically sound piece of scientific research with data that supports the conclusions. Experiments must have been conducted rigorously, with appropriate controls, replication, and sample sizes. The conclusions must be drawn appropriately based on the data presented.

Reviewer #1: Yes

2. Has the statistical analysis been performed appropriately and rigorously?

Reviewer #1: Yes

3. Have the authors made all data underlying the findings in their manuscript fully available?

The [PLOS Data policy](http://www.plosone.org/static/policies.action#sharing) requires authors to make all data underlying the findings described in their manuscript fully available without restriction, with rare exception (please refer to the Data Availability Statement in the manuscript PDF file). The data should be provided as part of the manuscript or its supporting information, or deposited to a public repository. For example, in addition to summary statistics, the data points behind means, medians and variance measures should be available. If there are restrictions on publicly sharing data—e.g. participant privacy or use of data from a third party—those must be specified.

Reviewer #1: No

***Response****: All study data (interviews with participants) are available in the manuscript and can be accessed.*

4. Is the manuscript presented in an intelligible fashion and written in standard English?

PLOS ONE does not copyedit accepted manuscripts, so the language in submitted articles must be clear, correct, and unambiguous. Any typographical or grammatical errors should be corrected at revision, so please note any specific errors here.

Reviewer #1: Yes

5. Review Comments to the Author

Please use the space provided to explain your answers to the questions above. You may also include additional comments for the author, including concerns about dual publication, research ethics, or publication ethics. (Please upload your review as an attachment if it exceeds 20,000 characters)

Reviewer #1: This manuscript is well-written. To improve the methodological part, the following are suggested:

1- Theme network resulting from three-stage coding

***Response****: Very thanks for your valuable comment.* *We hope that the answer is clear and can answer the question of the honorable reviewer.*

*In this study, the content analysis(CA) as a qualitative descriptive approach is used. Thematic analysis(TA) and one of the TA related to it, i.e. theme networking, have not been used. Although the boundaries between these two approaches are not clear and they are often used interchangeably, and there is still confusion about their similarities and differences.*

*As a point of difference, within TA, a theme is considered to be latent content, but CA researchers are free to decide between the level of data analysis when developing the category or the theme. One of the first decisions that should be taken when conducting CA is whether to concentrate analysis on the manifest or latent content of data. In this study, we concentrate on manifest (developing categories). While the thematic analysis researcher considers both latent content as theme and manifest content as category in data analysis, the content analyst chooses between them before proceeding to the higher levels of data analysis. A category is an idea that is directly expressed in the text, this is what we were looking for in this study, and we have progressed to the stage of category development.*

*Also, as dear reviewer pointed out encouraging creativity in findings is a common aspect of data analysis in both CA and TA. In other words, creativity, intuition and innovation for presenting the result in terms of a story line, a map, or model are crucial to data analysis. Drawing a thematic map or the visual presentation of themes, codes, and their relationships is a characteristic of data analysis in thematic analysis. In this study, we have used the story line to present the results*.

*References:*

*Vaismoradi, M., Turunen, H., & Bondas, T. (2013). Content analysis and thematic analysis: Implications for conducting a qualitative descriptive study. Nursing & health sciences, 15(3), 398-405.*

*Vaismoradi, M., Jones, J., Turunen, H., & Snelgrove, S. (2016). Theme development in qualitative content analysis and thematic analysis. ‏*

*Vaismoradi, M., & Snelgrove, S. (2019). Theme in qualitative content analysis and thematic analysis. In Forum Qualitative Sozialforschung/Forum: Qualitative Social Research (Vol. 20, No. 3). DEU. ‏*

2- Table of agreement between two coders or calculation of kappa coefficient.

***Response****: The value of kappa coefficient was added*.

3- The interview questions should come in the tools and methods section.

***Response****: Corrections were made.*

6. PLOS authors have the option to publish the peer review history of their article ([what does this mean?](https://journals.plos.org/plosone/s/editorial-and-peer-review-process#loc-peer-review-history)). If published, this will include your full peer review and any attached files.


If you choose “no”, your identity will remain anonymous but your review may still be made public.
